# Supplementary material for: KLF5 Is Crucial for Androgen-AR Signaling to Transactivate Genes and Promote Cell Proliferation in Prostate Cancer Cells
Source: Cancers (Basel). 2020 Mar 21;12(3):748. doi: 10.3390/cancers12030748 (PMC7140031; doi:10.3390/cancers12030748)
Supplement: Supplementary file 1 [file cancers-12-00748-s001.pdf]

Supplementary Figure 1. Unprocessed blot images for western blotting results.

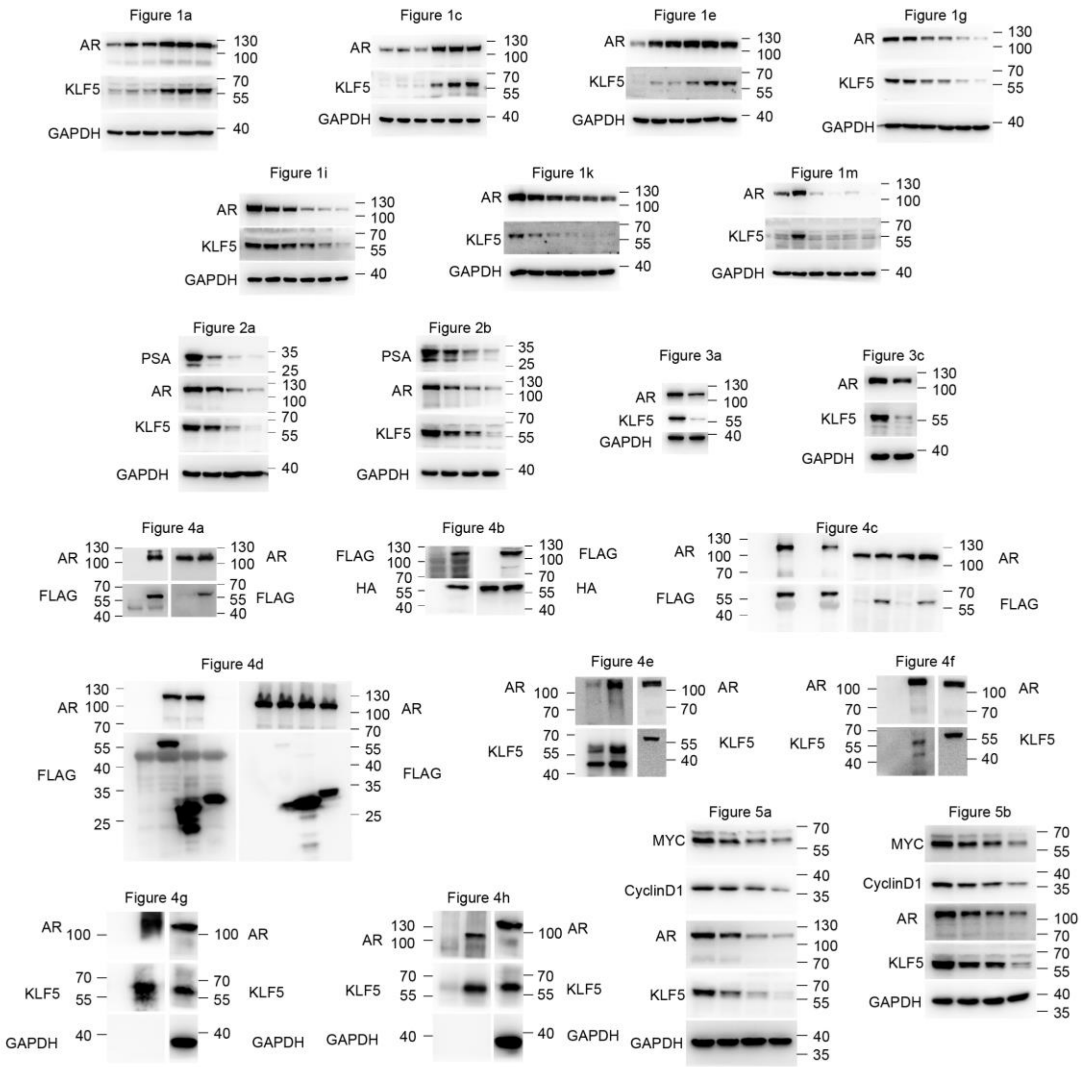

Supplementary Table 1. Primer sequences used in various PCRs in the study.

Gene name	Forward (5′ to 3′)	Reverse (5′ to 3′)
**For real time qPCR:**		
KLF5	ACTGCCCTCGGAGGAGCTGG	ATGCTCTGAAATTATCGGAACTG
AR	TCCATCTTGTCGTCTTCGGAA	GGGCTGGTTGTTGTCGTGT
PSA	GTGTGTGGACCTCCATGTTATT	TGCCCCATGACGTGATACCT
TMPRSS2	GCAGTGGTTTCTTTACGCTG	CCGCAAATGCCGTCCAATG
FKBP5	AATGGTGAGGAAACGCCGATG	TCGAGGGAATTTTAGGGAGACT
CyclinD1	CAATGACCCCGCACGATTTC	CATGGAGGGCGGATTGGAA
MYC	GTCAAGAGGCGAACACACAAC	TTGGACGGACAGGATGTATGC
GAPDH	GGTGGTCTCCTCTGACTTCAACA	GTTGCTGTAGCCAAATTCGTTGT
**For ChIP-qPCR:**		
A region	GCTCTTATCAGTCCT	GCTCCAGATTTCCTG
B region	CCTGGCGCCTAAACC	TCCCACCTCCTTTTC
C region	AGGCAAGGAGGCCGG	AGCGGCTAGCTCGGC
B1 region	TAGGCAGGCGTTAGCGC	TCTCCCCTCCCCTCACCG
B2 region	AAAAGGAAAGGGGAGGG	TCCCACCTCCTTTTCCCT
MYC	ACGGCCGACCAGCTGGAGAT	TCGTCGTCCGGGTCGCAGAT
CyclinD1	CGTGCCCGTGTGCATGTCCT	GTGGCCTTTCCCGACCCTGC
PSA promoter	TCTGCCTTTCTCCCCTAGAT	AACCTTCATTCCCCAGGACT
TMPRSS2 enhancer	TGGAGCTAGTGCTGCATGTC	CTGCCTTGCTGTGTGAAAAA
FKBP5	GGTTCCTGGGCAGGAGTAAG	AACGTGGATCCCACACTCTC
**For gene cloning**		
AR	CTCGAGTCTATACACATTATGTCT	CGGCGGCTCGAAGCCGCTAAGCTT
AR1	CTCGAGTCTATACACATTATGTCT	AACCTTAGATCCGTCATAAAGCTT
AR2	CTCGAGCAGCAAGTATCTGCTGGC	CGGCGGCTCGAAGCCGCTAAGCTT
